# Cognitive Status in People With Epilepsy in the Republic of Guinea: A Prospective, Case–Control Study

**DOI:** 10.1002/acn3.70282

**Published:** 2025-12-16

**Authors:** Maya L. Mastick, Cheick O. Soumah, Malé Doré, Oumar Mara, Desiré Neldje, Fodé A. Cissé, Toure M. Lamine, Aminata Diallo, Seungwon Lee, Siddharth Satish, Alexander J. X. Chen, Alice Liu, Nomin Enkhtsetseg, Farrah J. Mateen, The Guinea Epilepsy Project

**Affiliations:** ^1^ Harvard College Cambridge Massachusetts USA; ^2^ Department of Neurology Massachusetts General Hospital Boston Massachusetts USA; ^3^ Ignace Deen Hospital Conakry Guinea; ^4^ University of Gamal Abdel Nasser Conakry Guinea; ^5^ Harvard T.H. Chan School of Public Health Boston Massachusetts USA; ^6^ Davee Department of Neurology, Feinberg School of Medicine Northwestern University Chicago Illinois USA

**Keywords:** Africa, case–control studies, cognition, epilepsy, Montreal Cognitive Assessment, neuropsychological tests

## Abstract

**Objective:**

People with epilepsy (PWE) may experience cognitive deficits but fail to undergo formal evaluation. This study compares cognitive status between PWE and healthy controls in the West African Republic of Guinea.

**Methods:**

A cross‐sectional, case–control study was conducted in sequential recruitment phases (July 2024–July 2025) at Ignace Deen Hospital, Conakry. Adult (≥ 18 years) PWE enrolled consecutively, excluding those with a seizure within the past 24 h. Controls were healthy adults accompanying PWE at the hospital. Cognitive status was assessed with the Montreal Cognitive Assessment (MoCA) in French or translated into the patient's preferred language (Pular, Susu, Maninka, Kissi) as needed.

**Results:**

We enrolled 100 PWE (mean age 30.4 years, range 18–71, SD = 12.0) and 100 controls (mean age 39.4 years, range 19–70, SD = 12.3). Although 93% of PWE had previously used anti‐seizure medications (ASMs), only 85% were currently receiving treatment and 50% reported interrupted access to ASMs, primarily due to cost barriers. The mean MoCA score of controls (21.8, SD = 4.9) was higher than that of PWE (17.9, SD = 6.1; mean difference −4.2, 95% CI [−5.6, −2.8], SE = 0.69, *p* < 0.001), adjusted for education level, sex, age, and language. Participants who attended lower secondary, upper secondary, or university education scored 4.9, 5.3, and 8.3 points higher, respectively, than those with no school or primary education (all *p* < 0.001). Speaking an indigenous language was on average associated with a 2.5‐point decline in MoCA scores (95% CI [−3.8, −1.2], SE = 0.65, *p* < 0.001).

**Interpretation:**

PWE in Guinea demonstrated significantly lower cognitive performance on the MoCA compared to healthy controls, even after adjusting for covariates.

## 1 Introduction

Epilepsy affects over 50 million people globally, approximately 80% of whom reside in low‐ and middle‐income countries (LMICs) [1]. While the worldwide mortality rate associated with epilepsy has declined between 1990 and 2021, epilepsy‐related mortality in the Republic of Guinea, a low‐income country (LIC) in West Africa, has increased by 16% [2]. In addition to mortality, epilepsy can cause structural and functional changes in the brain that manifest as cognitive and neuropsychological disorders [3]. Recurring, prolonged seizure activity alters synaptic excitation and inhibition patterns in the hippocampus and neocortex and impairs long‐term potentiation, both of which are critical for memory and learning [4]. If seizures are not adequately managed, the resulting pathophysiological changes may lead to persistent cognitive deficits, including memory impairment, attention problems, and mental slowness [5, 6]. Multiple mechanisms may account for cognitive deficits. Hippocampal sclerosis is associated especially with temporal lobe epilepsy and causes neuronal loss and gliosis, leading to hippocampal atrophy and cognitive decline over time [7]. Post‐ictal hypoxia may also lead to cognitive dysfunction [8]. Epileptic activity can disrupt normal brain development and established neural circuitry, leading to persistent cognitive deficits and even epileptic encephalopathies [4, 9].

As compared to having remote (> 12 months prior) or no seizures, seizures occurring within the past year are associated with 2.1 times higher risk of cognitive impairment in otherwise cognitively healthy adults [10]. In LICs such as Guinea, the World Health Organization estimates that as many as 75% of PWE do not receive treatment due to factors such as limited access to anti‐seizure medications (ASMs) and qualified healthcare professionals [1, 11, 12]. The resulting burden of active epilepsy underscores the need to investigate cognitive status in these populations.

The Montreal Cognitive Assessment (MoCA) was originally developed in Canada as a screening tool for mild cognitive impairment (MCI) and early dementia in older adults who had on average 13 years of formal education [13]. It has since been validated in PWE in high‐income and high education settings [14, 15]. The MoCA's utility to diagnose cognitive deficits among poorly educated older adults with MCI irrespective of literacy has also been demonstrated [16], but its cultural appropriateness and feasibility for use in younger, lower‐literacy PWE in Guinea remains unclear. We hypothesized that PWE would have lower total cognitive MoCA scores than healthy controls for both sexes and that the MoCA would be a feasible, informative tool for assessing cognitive status in this setting. Our objectives were to evaluate the cognitive status in PWE using the MoCA by comparing results with healthy controls and to qualitatively assess the tool's applicability. Cognitive data are analyzed in relation to clinical and sociodemographic variables to better characterize the cognitive burden of epilepsy in this understudied context.

## 2 Methods

### 2.1 Ethics Statement

The institutional review boards of Mass General Brigham (Boston, MA) and Ignace Deen Hospital (IDH) (Conakry, Republic of Guinea) approved this study. Participants provided individual written or, if illiterate, thumbprint consent. All study materials were translated into the participant's preferred language by a Guinean physician who was fluent in the language needed. All study procedures conformed to the World Medical Association Declaration of Helsinki.

### 2.2 Setting

The Republic of Guinea is a low‐income country located in West Africa with a population of approximately 14.4 million as of 2025 [17]. It ranks 179th out of 193 countries on the United Nations Human Development Index [17]. The capital city, Conakry, is the largest urban center, with an estimated population of 2 million people [18]. IDH is located in Conakry and is one of the few hospitals in Guinea; all practicing neurologists in the country are stationed there [19]. Although French is the official language, Guinea is a linguistically diverse nation, with most of the population speaking at least one indigenous language. The government officially recognizes six indigenous languages in addition to French: Pular (or Fula), Maninka, Susu, Kissi, Kpelle, and Toma [20]. According to the 2014 census, Pular is the most widely spoken indigenous language, spoken by 35% of the population, followed by Maninka (25%), Susu (18%), and Kissi (4%) [20].

Guinea's education system includes 6 years of primary school, 4 years of lower secondary school, and 3 years of upper secondary education, and then the option of university [21]. However, only two‐thirds of Guinean children complete primary school, with an average of 7 years of schooling in total [21]. There is a significant gender gap in education in Guinea, as only 31% of girls enroll in secondary education compared to 41% of boys [21]. The last reported literacy rate was 61.2% for males aged 15 years and above, compared to 31.3% of females of the same age in 2021 [22].

### 2.3 Recruitment

Inclusion criteria for PWE were a history of two or more unprovoked seizures, residence in Guinea, and age > 18 years. Exclusion criteria for PWE included having a seizure in the past 24 h, head imaging results with evidence of structural intracranial conditions that could independently affect cognition (e.g., stroke, brain tumor, hydrocephalus, neurocysticercosis), or known competing conditions (e.g., HIV/AIDS, inborn errors of metabolism). For healthy controls, exclusion criteria were: (1) has a medical condition (e.g., diabetes, stroke, cancer, etc.); (2) uses illicit drugs/alcohol; or (3) has been hospitalized in the past 30 days. Participants were enrolled on a consecutive basis at the IDH Department of Neurology outpatient clinic during routine clinical visits, including medical appointments, admissions, and medication refills. Many PWE traveled from far across the country to receive care. Healthy controls were recruited from relatives or friends accompanying PWE in the hospital area. Recruitment was conducted by Guinean neurologists in three sequential recruitment phases from July 2024 to July 2025. Cases and controls were enrolled contemporaneously. Each participant was reimbursed 90,000 GNF (approximately 10 USD).

### 2.4 Cognitive Testing

Cognition was assessed using the 30‐point MoCA, with lower scores indicating lower cognitive status. The MoCA was selected for its ease of administration and because it has been studied across diverse populations. The French version was obtained from the official website, and local physicians translated it into Susu, Pular, Kissi, or Mainka when needed. Standardized instructions were read aloud before testing began, and participants with ≤ 12 years of formal education received one additional point on their final score. MoCA subsections include visuospatial/executive, naming, attention, language, abstraction, delayed recall, and orientation domains. The memory index score (MIS) is calculated separately from the MoCA score by adding the total number of words remembered across free delayed, category‐cued, and multiple choice‐cued recall categories.

### 2.5 Data Collection

Participants completed structured questionnaires that collected self‐reported demographics, clinical information, and qualitative experiences regarding epilepsy prior to taking the MoCA (Appendix III). We did not formally assess literacy among participants at the time of enrollment. Instead, participants were asked whether they could read and write well enough to complete the questionnaires; trained study staff read aloud and recorded responses for those who could not. Qualitative data were obtained through open‐ended questions exploring perceived causes and impacts of epilepsy. For participants with limited literacy, trained research staff transcribed verbal responses; otherwise, participants recorded their own responses in writing. Surveys and assessments took place outside of clinical visits in a separate examination room free from distractions. All data were entered into a secure online database for analysis.

Investigators documented observations during testing, noting hesitation or misunderstandings, especially for MoCA tasks that may have been affected by cultural context, language, or education. The concepts of “montre” (wristwatch) vs. “horloge” (clock) and “tigre” (tiger) vs. “lion” were not always semantically or culturally distinct for participants, so both substitutions were accepted with the correct reasoning and execution (e.g., drawing an analog wristwatch at the correct time instead of an analog clock). Any other applicability issues were noted during test administration and used to contextualize results and assess potential test applicability issues.

### 2.6 Data Analysis

All analyses were conducted using the programming language R (Vienna, Austria). Participants with missing survey data were included if the MoCA and primary variables of interest (age, sex, education, and language) were fully completed. Descriptive statistics were used to summarize demographic and cognitive test variables by group. Between‐group differences in MoCA scores were assessed using multiple linear regression, adjusting for age, sex, education level, and language. Linear mixed‐effects models with group‐domain interactions were used to examine subdomain differences. Pairwise post hoc comparisons used Bonferroni correction, and all models were evaluated for assumptions of normality and homoscedasticity when relevant. A significance threshold of *α* < 0.05 was applied throughout. To match controls to cases 1:1, we randomly selected a subset of controls using an online random number generator to reduce potential selection bias. Sensitivity analyses were performed post hoc, excluding those > 60 years old, seizure‐free for > 12 months, and with no or low literacy levels to assess the robustness of findings; these results were consistent with primary analyses.

### 2.7 Variables

The primary outcome variable was the education‐adjusted total MoCA score, which was obtained through the administration of the MoCA by trained research staff. Secondary outcome variables were domain‐specific MoCA subscores. The primary independent variable was group status (epilepsy vs. control). Covariates included education level (categorized as no school, “école primaire,” “college,” “lycée,” and “université” which correspond to the primary, lower secondary, upper secondary, and university levels of education in Guinea), age (in years), sex (male/female), and primary language (separated into two categories: non‐indigenous and indigenous). All variables were assessed in both epilepsy and control groups using the same tests and procedures to ensure comparability. Results reporting adhered to STROBE guidelines for a case–control study [23].

## 3 Results

### 3.1 Participants

**FIGURE 1:**
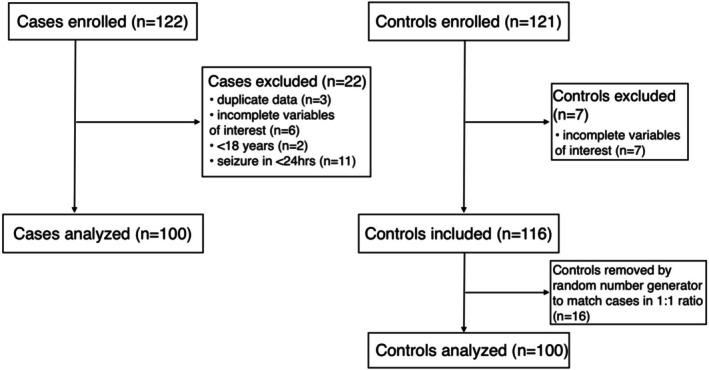
Participant flow chart indicating sample size, exclusion, and retention rates.

A total of 122 cases and 121 controls were enrolled in the study. After checking survey responses, 22 PWE were excluded due to duplicate data (*n* = 3), incomplete data (*n* = 6, missing: age = 1, education = 2, language = 2, MoCA = 1), or violating inclusion criteria in a survey response (*n* = 13). Of those who violated inclusion criteria, 2 PWE were removed since they were < 18 years by their reported date of birth, and 11 were removed because they had reported a seizure in the past 24 h. 7 controls were excluded due to incomplete data (missing: education = 1, MoCA = 6). To address the imbalance in cases and controls, a subset of 100 was randomly selected from the larger pool of eligible controls (*n* = 114). The analytic sample included 100 PWE and 100 controls (Figure 1).

### 3.2 Cases

Out of 100 PWE, the mean age was 30.4 years (range 18–71, standard deviation (SD) 12.0) at enrollment and 21.1 years (range 7 months–71 years, SD 14.3) at epilepsy diagnosis. There were only seven participants > 60 years old (2 cases, 5 controls) and five > 65 years old (two cases, three controls). 43% were female and 57% were male. Educational attainment included: no schooling (*n* = 10), primary (*n* = 16), lower secondary (*n* = 23), upper secondary (*n* = 30), and university‐level (*n* = 21). French was the preferred language for 47 PWE, followed by Pular (*n* = 28), Susu (*n* = 12), Maninka (*n* = 10), Kissi (*n* = 2), and English (*n* = 1) (Table 1). The mean MoCA score for cases was 17.9 (SD 6.1), with a median of 19 (interquartile range (IQR) 22–14.75).

**TABLE 1:** Demographic Information of all Participants.

Group	Control (*n* = 100)	Cases (PWE) (*n* = 100)
Age, years (y), mean (SD)	39.4 (12.3)	30.4 (12.0)
Range, y	19–70	18–71
18–24	15	46
25–29	16	12
30–34	11	14
35–39	13	12
40–44	11	4
45–49	16	4
50–54	10	6
55–59	3	4
60–64	2	4
65+	3	2
Sex, *n*		
Male	43	57
Female	57	43
Highest level education, *n*		
No school	18	10
Primary school	7	16
Lower secondary school	15	23
Upper secondary school	23	30
University	37	21
Preferred language, *n*		
French	39	47
Indigenous	61	52
Pular	29	28
Susu	15	12
Maninka	11	10
Kissi	5	2
Guéré	1	0
English	0	1
Unemployed or not in school, *n*	0	18
Employed or in school	100	82
Literate, *n*	95	95
No literacy	4	3
Low literacy	1	2

Nearly all (93%, *n* = 93) of PWE were diagnosed by a physician, few (*n* = 2) were diagnosed by a traditional healer, and the rest (*n* = 5) were unsure. All were evaluated by a study team neurologist. PWE had an average of 6.2 seizures in the past month (median 2.5, IQR 3, SD 34.3). 93% of PWE had previously taken ASMs, including levetiracetam (*n* = 40), carbamazepine (*n* = 40), sodium valproate (*n* = 10), phenobarbital (*n* = 7), clobazam (*n* = 5), lamotrigine (*n* = 2), topiramate (*n* = 1), phenytoin (*n* = 1), and clonazepam (*n* = 1). However, 85% were currently receiving ASM treatment, and only 50% had consistent access to medications. Interruptions or arrests in treatment were primarily because PWE ran out of donated medications and could not access or afford to buy more. Most (*n* = 78) were on monotherapy, but four individuals were on more than one ASM, including two on dual and two on triple therapy. Additional epilepsy information of PWE can be found in Table 2.

**TABLE 2:** Epilepsy Information of PWE (*n* = 100).

Age of epilepsy diagnosis, y, mean (standard deviation)	21.1 (14.3)
Diagnosed by doctor, *n*	93
Diagnosed by traditional healer, *n*	2
Unknown, but evaluated by study team	5
Lifetime seizure frequency, *n*	
> 100	11
50–99	4
10–49	8
2–9	7
“Many,” nonspecific	47
Not specified	23
First seizure occurrence, age, y, mean (standard deviation)	16.6 (14.6)
Last seizure occurrence, *n*	
Within the last week	37
Within the last month	31
Within the last year	19
Longer than 1 year ago	8
Unknown, but not < 24 h	5
Do any of these elements contribute to your seizures?	
Infections/fever	24
Lack of sleep	43
Flashing lights	7
Stress	43
Stopping medication (forget to take, run out, etc.)	37
Current medications, *n*	
Monotherapy (*n* = 81)	
Phenobarbital	4
Carbamazepine	33
Sodium valproate	3
Levetiracetam	37
Lamotrigine	2
Topiramate	1
Phenytoin	1
Polytherapy (*n* = 4)	
Carbamazepine/clobazam	2
Carbamazepine/levetiracetam/sodium valproate	1
Phenobarbital/sodium valproate/clonazepam	1
Traditional treatment, *n*	
Current (*n* = 28)	
Unknown	2
Decoctions, drinks, water	17
Prayer	8
Talismans	4
Special diet	2
Other (e.g., inhaling smoke)	2
Stopped (*n* = 34)	
Unknown	22
Decoctions, drinks, water	9
Talismans	1
Other (e.g., oil, powder)	4
MRI brain performed, *n*	7
CT head performed, *n*	60
EEG brain performed, *n*	52
Family history of seizures, *n*	23
Head injury with loss of consciousness, *n*	20
Seizure symptoms, *n*	
Loss of consciousness	95
Fall with tonic clonic movements of the limbs	84
Fall without convulsion	15
Uncontrollable movement of a part of the body	47
Frozen gaze	59
Unusual or strange behavior	31
Communication with spirits	12
Unusual sensations (visual, auditory, tactile, etc.)	22
Tongue bite	58
Loss of urine	61
Drooling (salivation)	72
Growling/screaming/any other form of noise	30

Qualitatively, local physicians administering the MoCA noted that language tasks (especially word generation starting with the letter “T”) assume familiarity with alphabetic systems, making those sections difficult for participants with no or low literacy or those who spoke only indigenous languages to complete. When asked about their community's beliefs regarding the cause of epilepsy, 64 PWE reported supernatural causes (e.g., devil, God, sorcery), though only 13 PWE personally held these beliefs. Half (*n* = 50) reported not knowing the cause, while the rest of PWE left no answer or attributed epilepsy to factors including headaches, divorce, female genital mutilation, stroke, and vaccines. The most commonly noted impacts were disruptions of study or work, followed by social stigma. Regarding treatment, 64 PWE believed medications were more effective than traditional remedies; others were unsure (*n* = 5), believed they were equally effective or ineffective (*n* = 4), or did not respond. Only one PWE reported traditional treatments as more effective than ASMs.

### 3.3 Controls

**FIGURE 2:**
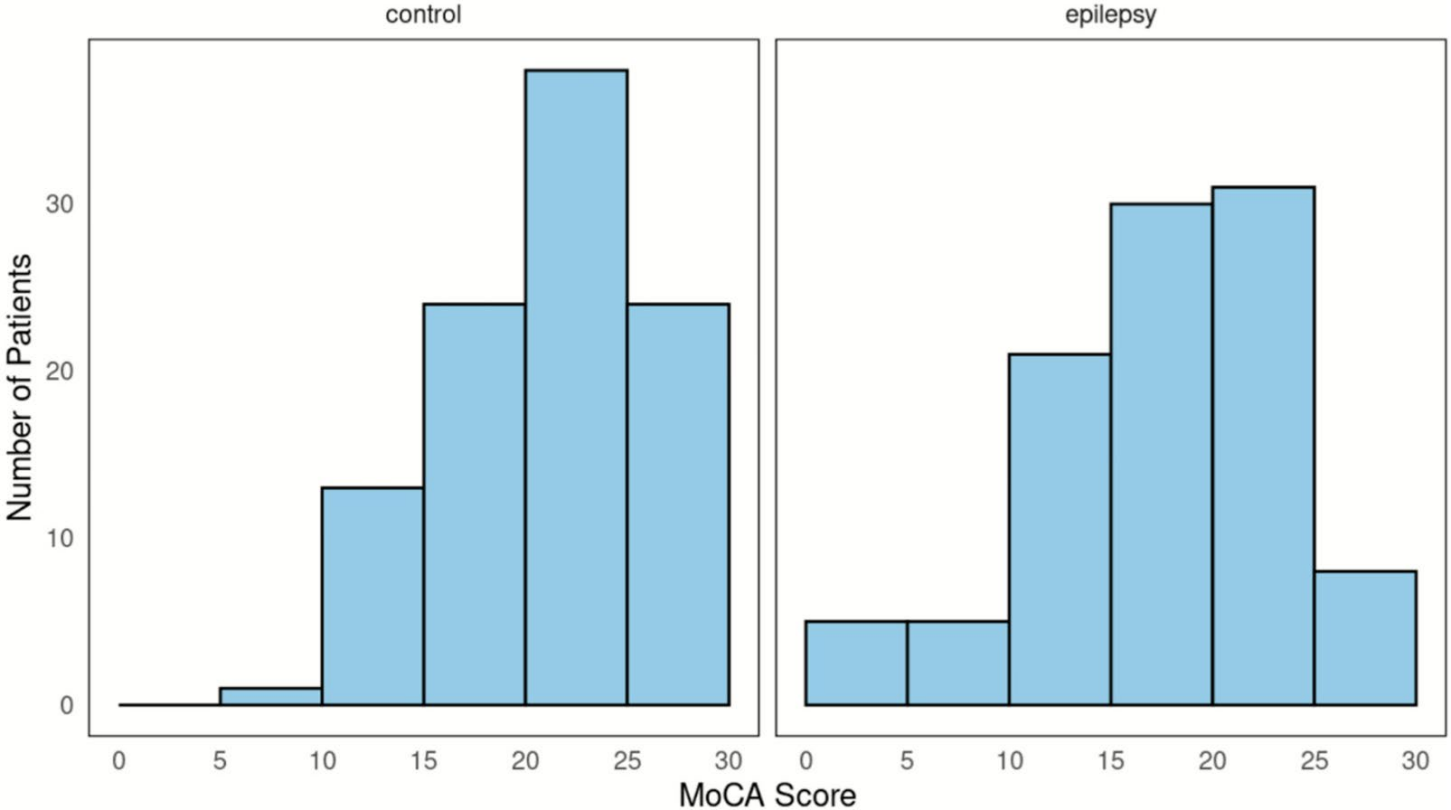
Histogram of MoCA scores by group. Bars represent the frequency of participants achieving each score. The conventional clinical cutoff for MCI is a MoCA score < 26.

Controls had a mean age of 39.4 years (range 19–70, SD 12.3). 43% were male and 57% were female. 7 controls had primary, 15 had lower secondary, 23 had upper secondary, and 37 had university‐level education, while 18 had no schooling at all. French was the preferred language of 39 controls, while 29 spoke Pular, 15 spoke Susu, 11 spoke Maninka, 5 spoke Kissi, and 1 spoke Guéré (Table 1). Compared with cases, controls were slightly older and more often female; the main imbalance was in education level, with 37% of controls having attended university versus 21% of PWE. The mean MoCA score for controls was 21.8 (SD 4.9), with a median of 23 (IQR 25–19). MoCA scores varied more widely among PWE (1–29) compared to controls (9–30) (Figure 2). Only 2 controls achieved the maximum possible score of 30 on the MoCA, whereas no PWE scored a 30. Additional MoCA details are included in Table 3.

**TABLE 3:** MoCA Scores of all Participants.

Group	Controls (*n* = 100)	Cases (PWE) (*n* = 100)
Total MoCA Score, *n*		
0–10	1	10
11–15	13	21
16–20	24	30
20–25	38	31
26–27	10	5
28–29	12	3
30	2	0
MoCA Score, mean (SD)	21.8 (4.9)	17.9 (6.1)
Male	23.6 (4.5)	19.1 (4.8)
Female	20.5 (4.9)	16.3 (7.2)
MoCA Score, median (IQR)	23 (25–19)	19 (22–14.75)
Male	24 (27–21)	19 (23–16)
Female	20 (23–16)	18 (21–12)
MoCA Sub‐scores, mean (SD)		
Visuospatial/executive (range 0–5)	2.3 (1.6)	1.6 (1.5)
Naming (range 0–3)	2.6 (0.68)	2.1 (1.0)
Attention (range 0–6)	4.5 (1.6)	3.5 (1.6)
Language (range 0–3)	1.9 (1.1)	1.4 (1.1)
Abstraction (range 0–2)	1.5 (0.81)	1.3 (0.93)
Delayed Recall (range 0–5)	3.0 (1.7)	2.2 (2.0)
Orientation (range 0–6)	5.7 (0.81)	5.2 (1.6)
Memory “MIS” (range 0–15)	10.6 (4.5)	8.4 (5.1)
Significant MoCA Point Differences between Cases and Controls (*p*‐value)	Estimated Difference	% of Sub‐score
Visuospatial/executive	−0.9 (*p* < 0.05)	18.5
Attention	−1.1 (*p* < 0.05)	18.5
Delayed Recall	−0.8 (*p* < 0.05)	15.6
Memory “MIS”	−1.9 (*p* < 0.0001)	12.6
MoCA Total (education‐adjusted)	−3.7 (*p* < 0.0001)	12.3

### 3.4 Inferential Analyses

To assess the contribution of demographic and clinical factors to overall MoCA scores, we fitted a multiple linear regression model including group status, education level, sex, language group, and age as potential predictors. Even after taking these covariates into account, PWE scored on average 4.2 points lower on the MoCA than controls (95% CI [2.8, 5.6]), SE = 0.69, F (8, 180 = 19.56, *p* < 0.001). This effect was large (Cohen's d = 0.97, SE = 0.17, 95% CI [0.64, 1.30]). The estimated adjusted mean MoCA score was 22.1 (SE = 0.5, 95% CI [21.1, 23.1]) for controls and 16.9 (SE = 0.46, 95% CI [16.0, 17.9]) for PWE. The model explained 44% of the variance in MoCA results (adj‐R^2 = 0.4413). Adjusted partial R^2 values suggest that epilepsy status accounts for 16.4%, education level for 27%, language preference for 7%, and age and sex each for 1.5% of this variance. Sensitivity analyses excluding participants > 60 years, with no seizures in the past 12 months, and with no or low literacy confirmed that cognitive differences existed in PWE (Appendix I).

Education level was a significant predictor of MoCA performance; those who had lower secondary education scored approximately 4.9* points higher, upper secondary scored 5.3* points higher, and university students scored 8.3* points higher than participants with no schooling (each *p < 0.001). There was no statistically significant difference between MoCA scores of cases versus controls for those who had attended no school or only primary school. Speaking an indigenous language was on average associated with a 2.5‐point decline in MoCA scores, 95% CI [−3.8, −1.2], SE = 0.65, *p* < 0.001. Age was a statistically significant factor but with modest impact, with each year predicting a linear 0.05‐point decrease on the MoCA, 95% CI [−0.1, −0.0001], *p* < 0.05. Men scored 1.2 points more on the MoCA than women on average, but this effect was not statistically significant (*p* = 0.057). ASM use was not significantly associated with MoCA performance. There were no interaction effects between group status and education, language, sex, or age.

**FIGURE 3:**
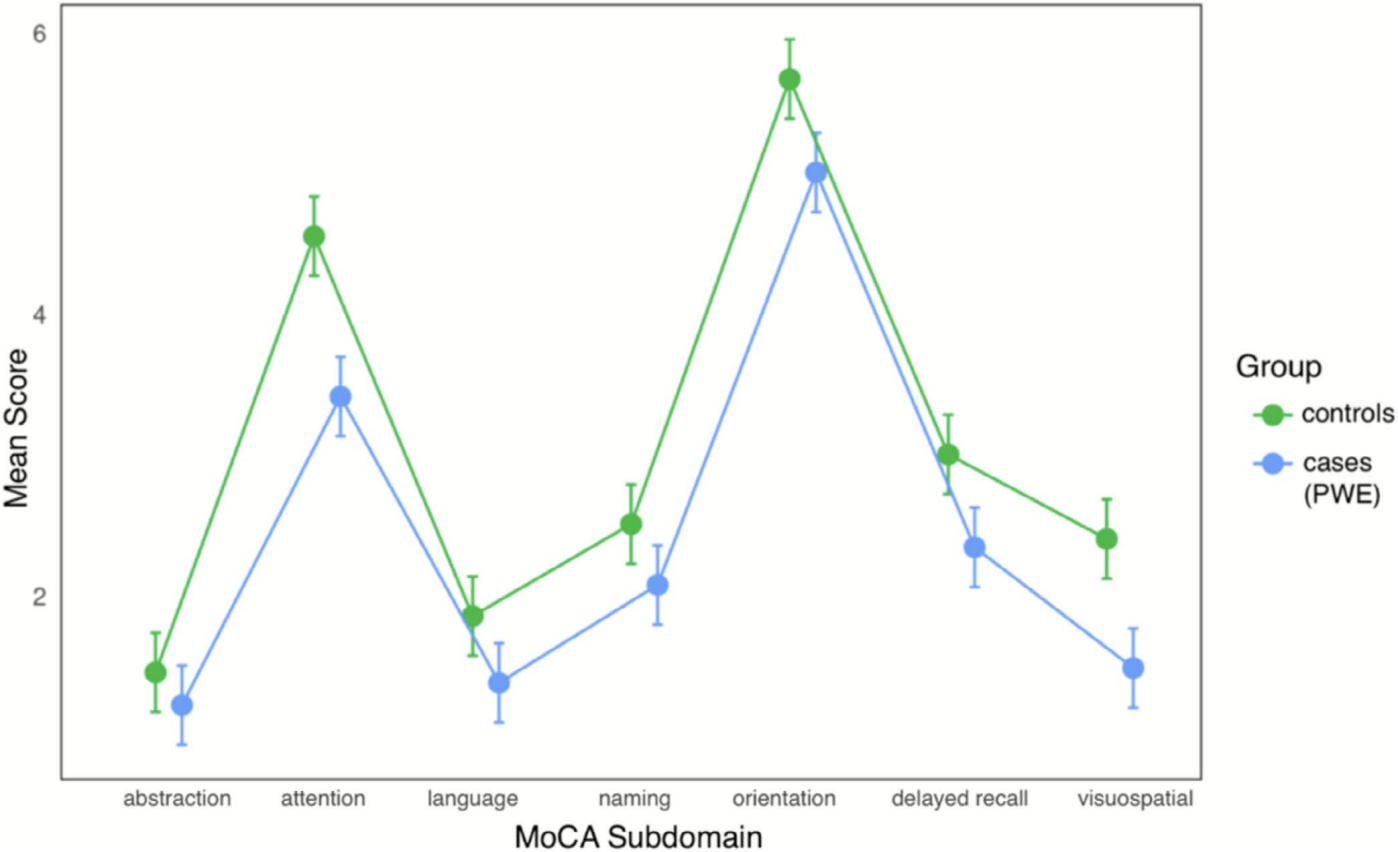
Mean MoCA scores by group, calculated from sums of the visuospatial/executive, naming, attention, delayed recall, and orientation subdomains. Error bars represent SE.

To evaluate how cognitive performance differed between PWE and healthy controls within the MoCA, we examined how MoCA subdomain scores varied across groups (Figure 3). Using a linear mixed‐effects model and adjusting for the above covariates, statistically significant differences were found in the visuospatial/executive, attention, memory, and delayed recall subdomains, where PWE scored 12%–19% lower than controls (Table 3). The naming, language, abstraction, and orientation MoCA sub‐scores did not differ significantly by group status (*p* > 0.05).

## 4 Discussion

This study gathered data on the cognitive burden experienced by PWE in the Republic of Guinea. Even after adjusting for covariates, PWE demonstrated poorer cognitive performance on the MoCA as compared to controls. The MoCA was selected due to its broad applicability across ages and conditions and for its previous validation in French and across the globe, including in African countries [24, 25, 26]. PWE's MoCA scores also spanned a larger point range than controls', reflecting greater variability in cognitive performance. This finding is consistent with prior studies, which reveal a link between chronic epilepsy and cognitive impairment [27, 28]. The official MoCA cutoff score for MCI is 26, although alternative cutoffs as low as 24 have been proposed to optimize MoCA sensitivity and specificity [14]. Most of our participants, both cases and controls, scored well below 26 (Table 3). While these results could indicate widespread cognitive deficits in Guinea, they may also reflect the impact of educational, cultural, and linguistic factors making it difficult for this population to achieve the maximal score. However, the MoCA shows separation and reflects multi‐domain subsections, allowing it to be useful in finding the spread and variation in cognitive performance. A more relevant test to Guinea is required and may come from neighboring countries. In the meantime, it remains noteworthy that the overall cognitive score for PWE was only around 18, indicating that brain health is not maximized in this vulnerable group.

**FIGURE 4:**
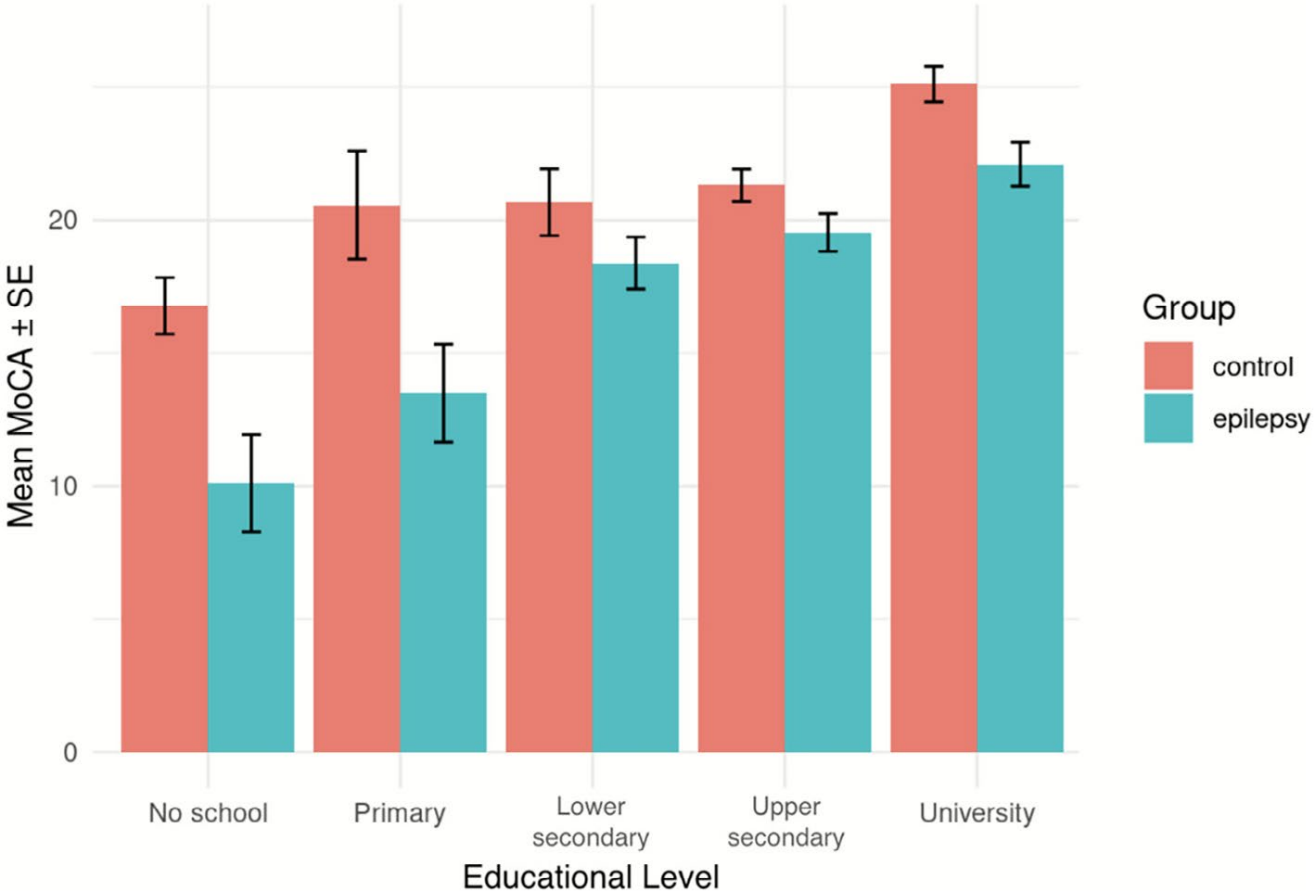
Mean MoCA scores displayed by epilepsy status and stratified by education. Error bars reflect standard error of the mean.

Education level was a strong predictor of MoCA performance. All participants scored significantly higher on the test with each successive level of schooling past primary education (Figure 4). The interplay of education and epilepsy is complex: PWE reported being forced to stop attending school early due to seizure‐related concerns, and this lack of education offers PWE fewer opportunities to build the cognitive reserve that can help buffer against the neurological effects of epilepsy [29]. Since PWE are less likely to attend and remain in school, the educational attainment of PWE in Guinea is known to be lower, even among PWE who seek the best available care [30]. Furthermore, language exposure in Guinea has historically been tied to education; post‐independence reform efforts in 1958 removed French from the Guinean school system and replaced it with indigenous languages, a decision later reversed in 1984 [31]. Access to education has also improved over time in Guinea, especially for women [22]. Therefore, our finding that age is significantly associated with MoCA scores may reflect not only age‐related cognitive decline but also residual differences in education between older and younger populations since most participants were not in high‐risk age ranges for neurodegenerative forms of MCI. While no statistically significant association between MoCA scores and sex was detected, we cannot exclude the possibility of type II error as this subgroup analysis may have been underpowered.

Shifts in the accessibility and content of education in Guinea may have influenced MoCA results. Qualitatively, examiners noted that the drawing tasks where participants had to connect letters and numbers in order (trails testing) draw a clock, and the word‐generation task beginning with a particular letter were particularly challenging (Appendix II). Those without a formal education struggled with all of these tasks, as some participants were unable to identify or write the necessary numbers and letters or hold a pen. However, participants who spoke an indigenous language with limited exposure to French were noted by Guinean physicians to struggle more with the MoCA word‐generation task, but not other language‐based tasks such as sentence repetition, than those who had any level of familiarity with French. This was interesting given that indigenous languages are primarily taught and spoken orally, and that 94% of our participants were literate, compared to just 35% of the Guinean population on average [17]. While we found no significant statistical association between age or education level and language, given the complexity of multilingualism in Guinea, these factors could have impacted language exposure and therefore MoCA scores. Guinean physicians who translated and administered the MoCA in indigenous languages suggested this task might have been improved by a phonemic rather than alphabetic cue, which would be recognizable even to participants with less French exposure or lower literacy levels.

Nearly two‐thirds of PWE reported that their family and/or community believed epilepsy was caused by sorcery or divine punishment, although significantly fewer PWE endorsed these explanations. This is similar to other LMICs, where superstitions regarding epilepsy can lead to social exclusion and worse socioeconomic status [32, 33]. PWE cited epilepsy‐related stigma as a barrier to education, employment, and marriage (especially for women). While we did not employ the stigma scale in the epilepsy questionnaire in this study, previous research shows that higher stigma is associated with higher seizure frequency and lower household wealth [34]. Educational attainment is also lower in poorer households, which are generally more rural and experience more stigma; likely, these variables are collinear [34]. Many PWE reported being removed from school due to head injuries or social concerns and rationing ASMs due to cost, sometimes leading to inconsistent treatment. Discontinuities in education and therapy can exacerbate cognitive deficits [35]. These findings underscore the importance of global “brain health” initiatives and better seizure control for PWE in Guinea, as many of these disparities can be rectified by earlier and more definitive access to ASMs.

The cognitive deficits observed among PWE were likely influenced by many factors: biological impacts of epilepsy (e.g., hypoxic events, hippocampal damage, frequent interictal activity, recurrent seizures), secondary effects of epilepsy (e.g., head traumas, medication side effects, education access, stigma), language barriers to the testing instrument, poverty, and testing willingness [36]. Consistent with prior research, these deficits were most pronounced in memory, attention, and visuospatial/executive domains [27, 37, 38]. Presumably, which cognitive processes are affected depends on the location of epileptic activity in the brain [38]. Seizure frequency specifically has been linked to problems with spatial orientation and memory, though some studies only report an association with one of the two [37, 39, 40, 41]. We found no relationship between seizure frequency and cognitive status, though inconsistent seizure reporting may have influenced these results. While certain ASMs, particularly phenobarbital and benzodiazepines, can impair cognition [42, 43], we observed no cognitive differences based on ASM use; however, we were unable to fully assess the impact of specific ASMs due to small sample sizes.

In addition to this limitation, we are unable to establish causality. Longitudinal studies including detailed seizure‐specific variables (e.g., frequency, duration, seizure type) and ASM data would strengthen inferences. While we attempted to account for the potential confounding effects of variables such as age and education, in practice it is not possible to fully control for these factors. In particular, the model likely incompletely adjusted for the disparity between the educational attainment of PWE and controls. However, this reflects the reality in Guinea, as PWE face multiple barriers to schooling and on average attain a lower educational level than their peers [30]. We did not assess language exposure beyond self‐reported preferred language, nor did we formally test participants' fluency in that language. Another limitation is that no back translation of the MoCA was completed into any of the Guinean indigenous languages. Future research should explicitly back translate and examine whether familiarity with Latin‐alphabet‐based languages correlates with MoCA performance independent of formal education. Selection bias may exist, as participants able or willing to travel to Conakry are unlikely to be representative of all PWE in Guinea. If present, this bias is probably positive, since our sample was more educated and had much better access to neurological care than the broader Guinean population.

Strengths of our study include the use of controls and enrollment of PWE in a resource‐limited setting with a high need for ASM treatment. Participants were overall young and had a high seizure burden. The study had a relatively well‐characterized, balanced sample in terms of language, education, age, and sex. Study assessments were performed by African physicians and translated into multiple indigenous languages. The MoCA has been validated in various populations and languages, but its applicability in populations with oral language traditions and high illiteracy rates has not been previously studied. While challenges in this study included visuospatial/executive, subtraction, language, and delayed recall tasks, the MoCA was overall valuable as a brief, multi‐domain evaluation for cognitive status. Future research should further assess the MoCA's applicability in Guinea and similar LMICs, adapt it for cultural and linguistic relevance, or consider supplementary tools like the Oxford Cognitive Screen, which rely less on literacy, to better characterize cognitive status in diverse populations [44].

## Author Contributions

Maya Mastick: data collection, data analysis, data interpretation, writing the first draft, and editing. Malé Doré, Cheick Ousmane Soumah, Oumar Mara, Desiré Neldje, Siddharth Satish, Seungwon Lee, Alexander Chen, Nomin Enkhtsetseg, Aminata Diallo: data collection. Alice Liu: study design. Fodé Abass Cissé: study supervision, data collection. Farrah J. Mateen: data interpretation, study design, writing, editing, obtaining funding, and study supervision.

## Funding

The authors have nothing to report.

## Conflicts of Interest

Maya Mastick and Seungwon Lee received MGH Community Engagement funding grants from the MGH Center for Global Health. No author has any conflicts of interest to disclose relevant to this article. All study procedures conformed to the World Medical Association Declaration of Helsinki; further information is included in the Methods section.

## Supporting information


**Data S1:** Supporting Information.

## Data Availability

The data that support the findings of this study are available on request from the corresponding author. The data are not publicly available due to privacy or ethical restrictions.

## References

[1] WHO , “Epilepsy,” (2025), https://www.who.int/news‐room/fact‐sheets/detail/epilepsy.

[2] V. L. Feigin , T. Vos , B. S. Nair , et al., “Global, Regional, and National Burden of Epilepsy, 1990–2021: A Systematic Analysis for the Global Burden of Disease Study 2021. Lancet,” Public Health 10, no. 3 (2025): e203–e227, 10.1016/S2468-2667(24)00302-5. 40015291 PMC11876103

[3] K. Van Rijckevorsel , “Cognitive Problems Related to Epilepsy Syndromes, Especially Malignant Epilepsies,” Seizure 15, no. 4 (2006): 227–234, 10.1016/j.seizure.2006.02.019. 16563807

[4] G. L. Holmes , “Cognitive Impairment in Epilepsy: The Role of Network Abnormalities,” Epileptic Disord Int Epilepsy J Videotape 17, no. 2 (2015): 101–116, 10.1684/epd.2015.0739. PMC5410366 25905906

[5] S. Landi , L. Petrucco , F. Sicca , and G. M. Ratto , “Transient Cognitive Impairment in Epilepsy,” Frontiers in Molecular Neuroscience 11 (2018): 458, 10.3389/fnmol.2018.00458. 30666185 PMC6330286

[6] C. Helmstaedter and J.‐A. Witt , “Epilepsy and Cognition—A Bidirectional Relationship?,” Seizure 49 (2017): 83–89, 10.1016/j.seizure.2017.02.017. 28284559

[7] M. Thom , S. Eriksson , L. Martinian , et al., “Temporal Lobe Sclerosis Associated With Hippocampal Sclerosis in Temporal Lobe Epilepsy: Neuropathological Features,” Journal of Neuropathology and Experimental Neurology 68, no. 8 (2009): 928–938. 19606061 10.1097/NEN.0b013e3181b05d67 PMC2723771

[8] J. S. Farrell , R. Colangeli , M. D. Wolff , et al., “Postictal Hypoperfusion/Hypoxia Provides the Foundation for a Unified Theory of Seizure‐Induced Brain Abnormalities and Behavioral Dysfunction,” Epilepsia 58, no. 9 (2017): 1493–1501. 28632329 10.1111/epi.13827

[9] M. Lenge , S. Balestrini , A. Napolitano , et al., “Morphometric Network‐Based Abnormalities Correlate With Psychiatric Comorbidities and Gene Expression in PCDH19‐Related Developmental and Epileptic Encephalopathy,” Translational Psychiatry 14, no. 1 (2024): 35. 38238304 10.1038/s41398-024-02753-x PMC10796344

[10] I. Zawar , J. Kapur , M. K. Mattos , C. M. Aldridge , C. Manning , and M. Quigg , “Association of Seizure Control With Cognition in People With Normal Cognition and Mild Cognitive Impairment,” Neurology 103, no. 6 (2024): e209820. 39173101 10.1212/WNL.0000000000209820 PMC11343585

[11] M. Jang , F. Sakadi , N. R. Tassiou , et al., “Impact of Poorly Controlled Epilepsy in the Republic of Guinea,” Seizure 61 (2018): 71–77. 30114675 10.1016/j.seizure.2018.07.018 PMC6168342

[12] C. K. Mbuba , A. K. Ngugi , C. R. Newton , and J. A. Carter , “The Epilepsy Treatment Gap in Developing Countries: A Systematic Review of the Magnitude, Causes, and Intervention Strategies,” Epilepsia 49, no. 9 (2008): 1491–1503. 18557778 10.1111/j.1528-1167.2008.01693.x PMC3573323

[13] Z. S. Nasreddine , N. A. Phillips , V. Bédirian , et al., “The Montreal Cognitive Assessment, MoCA: A Brief Screening Tool for Mild Cognitive Impairment,” Journal of the American Geriatrics Society 53, no. 4 (2005): 695–699. 15817019 10.1111/j.1532-5415.2005.53221.x

[14] A. Reyes , B. P. Hermann , D. Prabhakaran , et al., “Validity of the MoCA as a Cognitive Screening Tool in Epilepsy: Are There Implications for Global Care and Research?,” Epilepsia Open 9, no. 4 (2024): 1526–1537. 38874380 10.1002/epi4.12991 PMC11296095

[15] A. Novak , K. Vizjak , A. Gacnik , and M. Rakusa , “Cognitive Impairment in People With Epilepsy: Montreal Cognitive Assessment (MoCA) as a Screening Tool,” Acta Neurologica Belgica 123, no. 2 (2023): 451–456. 35925540 10.1007/s13760-022-02046-4

[16] P. Julayanont , S. Tangwongchai , S. Hemrungrojn , et al., “The Montreal Cognitive Assessment‐Basic: A Screening Tool for Mild Cognitive Impairment in Illiterate and Low‐Educated Elderly Adults,” Journal of the American Geriatrics Society 63, no. 12 (2015): 2550–2554. 26648041 10.1111/jgs.13820

[17] Nations U. Specific country data , “Human Development Reports,” (2025), https://hdr.undp.org/data‐center/specific‐country‐data.

[18] Conakry Population , “World Population Review,” (2025), https://worldpopulationreview.com/cities/guinea/conakry.

[19] P. Anand , “Global & Community Health: The Djina Disease,” Neurology 92, no. 15 (2019): 725–727. 30962298 10.1212/WNL.0000000000007274 PMC6511090

[20] Language data for Guinea , “Translators Without Borders,” (2025), https://translatorswithoutborders.org/language‐data‐for‐guinea/.

[21] UNESCO , “Guinea: Education Country Brief | International Institute for Capacity Building in Africa,” (2025), https://www.iicba.unesco.org/en/guinea.

[22] World Bank Gender Data Portal , “Guinea,” (2025), https://genderdata.worldbank.org/en/economies/guinea.

[23] STROBE , “Strengthening the Reporting of Observational Studies in Epidemiology,” (2025), https://www.strobe‐statement.org/checklists/.

[24] K. Dujardin , S. Duhem , N. Guerouaou , et al., “Validation in French of the Montreal Cognitive Assessment 5‐Minute, a Brief Cognitive Screening Test for Phone Administration,” Revue Neurologique (Paris) 177, no. 8 (2021): 972–979. 10.1016/j.neurol.2020.09.002 33478740

[25] U. Sodov , K. Zuunnast , H. Stefan , and T. Avirmed , “Can MOCA be Applied for Rough Cognitive Assessment in Patients With Epilepsy in Mongolia?,” Journal of Clinical Medicine 14, no. 10 (2025): 3372. 40429369 10.3390/jcm14103372 PMC12111925

[26] G. M. Masika , D. S. F. Yu , and P. W. C. Li , “Accuracy of the Montreal Cognitive Assessment in Detecting Mild Cognitive Impairment and Dementia in the Rural African Population,” Arch Clin Neuropsychol off J Natl Acad Neuropsychol 36, no. 3 (2021): 371–380. 10.1093/arclin/acz086 31942599

[27] C. E. Elger , C. Helmstaedter , and M. Kurthen , “Chronic Epilepsy and Cognition,” Lancet Neurology 3, no. 11 (2004): 663–672. 15488459 10.1016/S1474-4422(04)00906-8

[28] B. Hermann and M. Seidenberg , “Epilepsy and Cognition,” Epilepsy Curr 7 (2007): 151.x, 10.1111/j.1535-7511.2007.00151.x. 17304341 PMC1797884

[29] Y. Stern , “What Is Cognitive Reserve? Theory and Research Application of the Reserve Concept,” J Int Neuropsychol Soc JINS 8, no. 3 (2002): 448–460. 11939702

[30] W. Fitts , N. T. Rahamatou , C. F. Abass , et al., “School Status and Its Associations Among Children With Epilepsy in the Republic of Guinea,” Epilepsy Behav 97 (2019): 275–281. 31260925 10.1016/j.yebeh.2019.05.040 PMC6702082

[31] M. S. Diallo , “Basic Education Reforms in Guinea: Context and Concerns. West East,” J Soc Sci 8, no. 3 (2019): 34.

[32] B. H. M. de , “Epilepsy Stigma: Moving From a Global Problem to Global Solutions,” Seizure ‐ Eur J Epilepsy 19, no. 10 (2010): 630–636. 10.1016/j.seizure.2010.10.017 21075013

[33] B. Hermann and A. Jacoby , “The Psychosocial Impact of Epilepsy in Adults,” Epilepsy Behav 15, no. 1 (2009): S11–S16. 19318133 10.1016/j.yebeh.2009.03.029 PMC3680516

[34] D. R. Rice , F. A. Cisse , A. B. Djibo Hamani , et al., “Epilepsy Stigma in the Republic of Guinea and Its Socioeconomic and Clinical Associations: A Cross‐Sectional Analysis,” Epilepsy Research 177 (2021): 106770. 34619642 10.1016/j.eplepsyres.2021.106770 PMC8557132

[35] A. T. Berg , J. T. Langfitt , F. M. Testa , et al., “Global Cognitive Function in Children With Epilepsy: A Community‐Based Study,” Epilepsia 49, no. 4 (2008): 608–614. 18070088 10.1111/j.1528-1167.2007.01461.x

[36] A. Novak , K. Vizjak , and M. Rakusa , “Cognitive Impairment in People With Epilepsy,” Journal of Clinical Medicine 11, no. 1 (2022): 267. 35012007 10.3390/jcm11010267 PMC8746065

[37] B. P. Hermann , M. Seidenberg , C. Dow , et al., “Cognitive Prognosis in Chronic Temporal Lobe Epilepsy,” Annals of Neurology 60, no. 1 (2006): 80–87. 16802302 10.1002/ana.20872

[38] S. V. Thomas and A. Nair , “Confronting the Stigma of Epilepsy,” Annals of Indian Academy of Neurology 14, no. 3 (2011): 158–163. 22028525 10.4103/0972-2327.85873 PMC3200035

[39] A. D. Saint‐Martin , C. Seegmuller , R. Carcangiu , et al., “Cognitive Consequences of Rolandic Epilepsy,” Epileptic Disord Int Epilepsy J Videotape 3, no. 2 (2001): SI59–SI165. 11827848

[40] J. Weglage , A. Demsky , M. Pietsch , and G. Kurlemann , “Neuropsychological, Intellectual, and Behavioral Findings in Patients With Centrotemporal Spikes With and Without Seizures,” Developmental Medicine and Child Neurology 39, no. 10 (1997): 646–651. 9352724 10.1111/j.1469-8749.1997.tb07357.x

[41] Wiley Online Library , “Factors for Cognitive Impairment in Adult Epileptic Patients ‐ Wang ‐ 2020 ‐ Brain and Behavior,” (2025), 10.1002/brb3.1475. PMC6955925 31863643

[42] D. W. Loring and K. J. Meador , “Cognitive Side Effects of Antiepileptic Drugs in Children,” Neurology 62, no. 6 (2004): 872–877. 15037684 10.1212/01.wnl.0000115653.82763.07

[43] S. A. Stewart , “The Effects of Benzodiazepines on Cognition,” Journal of Clinical Psychiatry 66, no. 2 (2005): 9–13. 15762814

[44] N. Demeyere , M. J. Riddoch , E. D. Slavkova , et al., “Domain‐Specific Versus Generalized Cognitive Screening in Acute Stroke,” Journal of Neurology 263, no. 2 (2016): 306–315. 26588918 10.1007/s00415-015-7964-4 PMC4751179

