# Supplementary material for: Cognitive Status in People With Epilepsy in the Republic of Guinea: A Prospective, Case–Control Study
Source: Ann Clin Transl Neurol. 2025 Dec 16;13(5):994–1004. doi: 10.1002/acn3.70282 (PMC13161882; doi:10.1002/acn3.70282)
Supplement: Supplementary file 1 — Data S1: Supporting Information. [file ACN3-13-994-s001.docx]

**Appendix I –** Sensitivity Analyses of Cognitive Test Score Differences between Cases and Controls, based on Varying Participant Characteristics

|  | MoCA Score, mean (SD) | Sample Size | p-value | Case-Control MoCA Difference [95% CI] |
| --- | --- | --- | --- | --- |
| Main analysis  Controls  Cases | 21.8 (4.9)  17.9 (6.1) | 100  100 | p<.0001 | -3.95  [-6.02, -2.81] |
| Active epilepsy only  Controls  Cases | 21.4 (5.1)  16.8 (6.2) | 100  92 | p<.0001 | -4.24  [-5.66, -2.82] |
| Excluding >60 years  Controls  Cases | 21.7 (5.1)  17.3 (6.3) | 98  95 | p<.0001 | -4.29  [-5.94, -2.63] |
| +3 points / participant with no or low literacy  Controls  Cases | 22.0 (4.9)  18.1 (6.1) | 100  100 | p<.0001 | -4.28  [-5.65, -2.90] |

*Rationale:*

PWE with seizures >12 months prior (n=8) were excluded to determine if active epilepsy impacts cognition.

Participants >60 years (n=7: 2 controls, 5 cases) were excluded to reduce risk of Alzheimer-type mild cognitive impairment influencing results.

Participants with no or low literacy as determined by consent by fingerprint or physicians’ note had 3 points added to their score (n=10: 5 controls, 5 cases) since there were three MoCA points requiring knowledge of letters/words to reduce the risk of literacy confounding results.

*Results:* Removing older participants or restricting to active epilepsy did not meaningfully change the observed MoCA score differences. This indicates that the observed cognitive deficits in PWE are not driven by age-related decline or seizure recency in this study.

**Appendix II –** PWE drawings representing common visuospatial/executive scoring difficulties


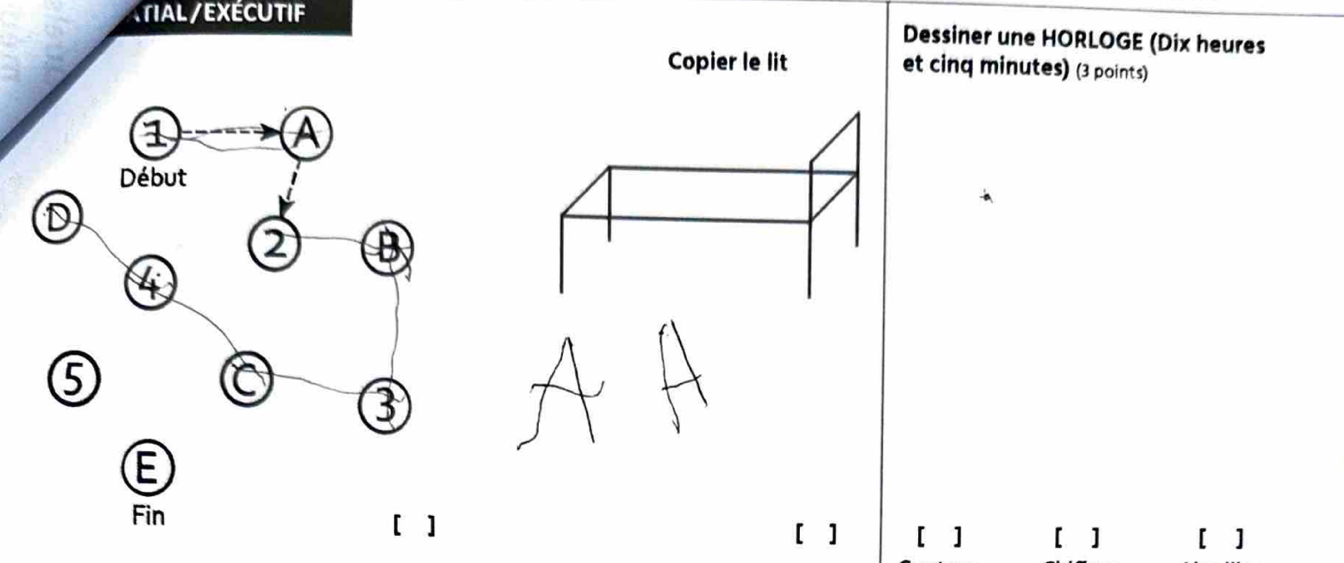


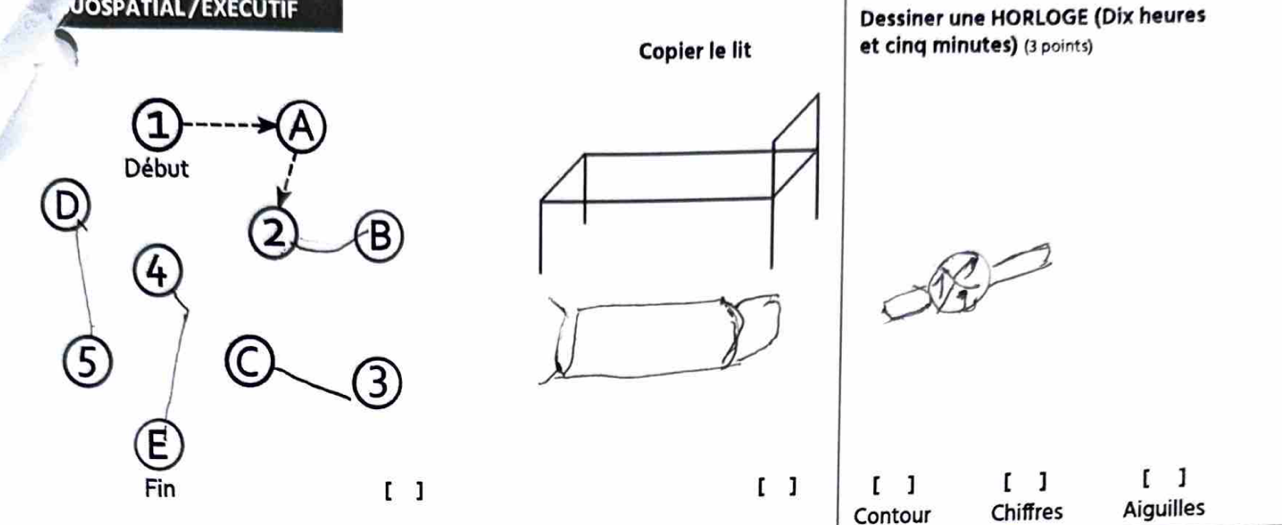


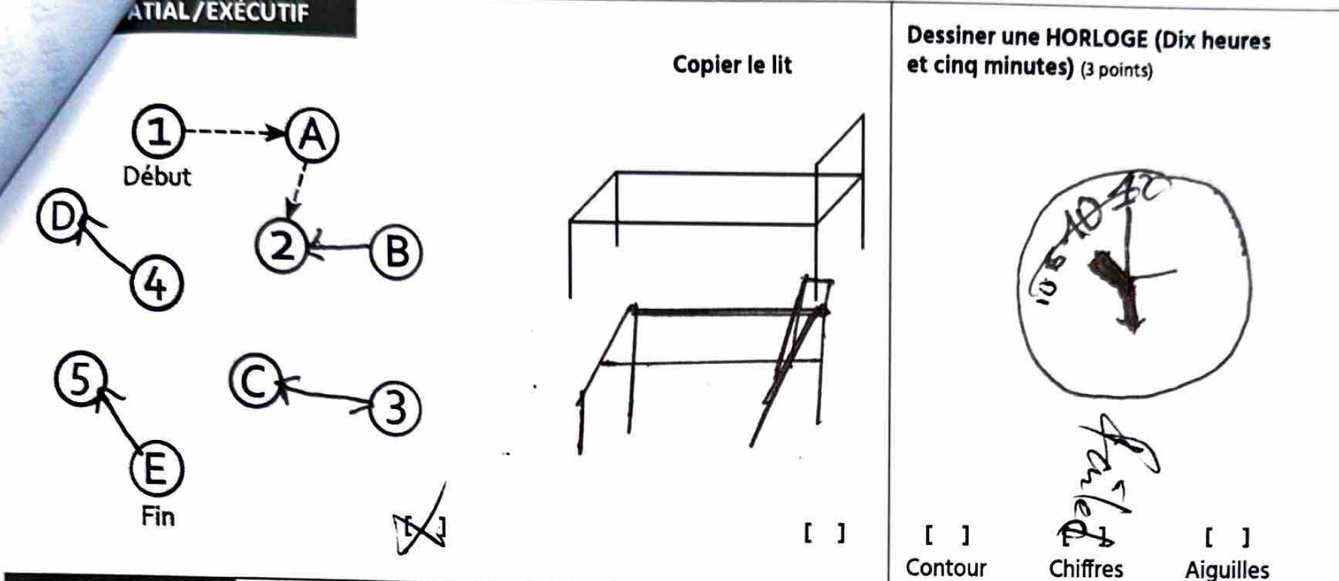


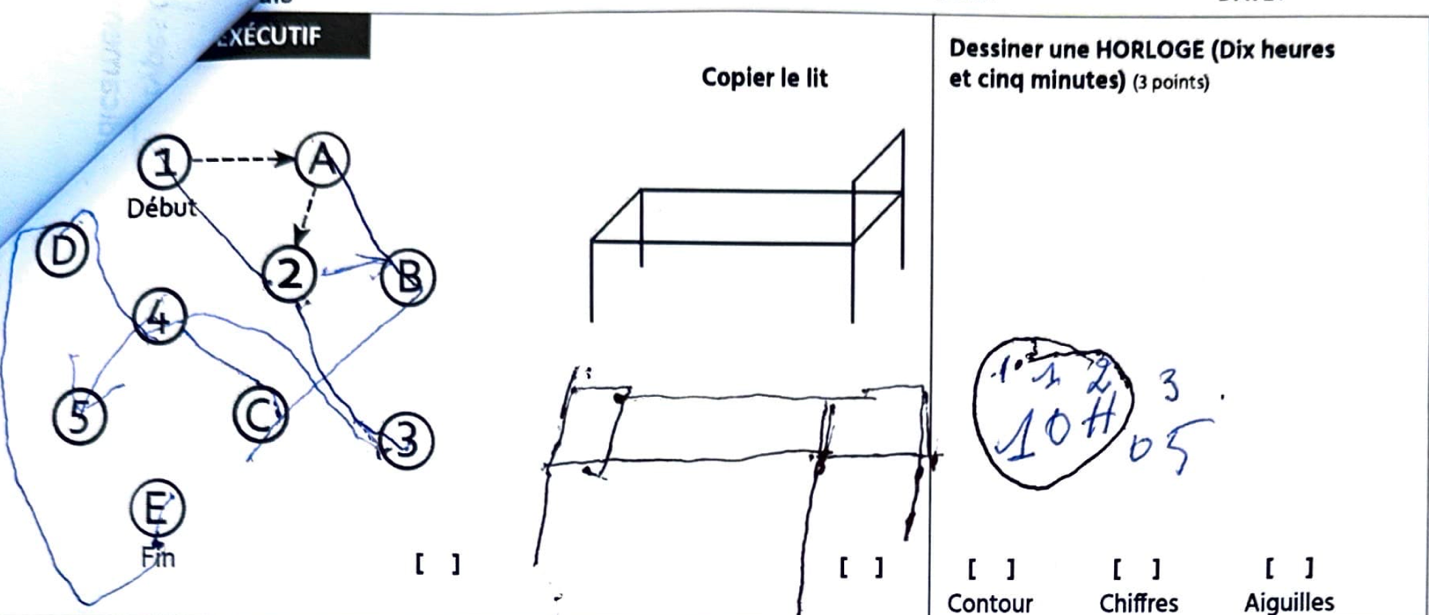


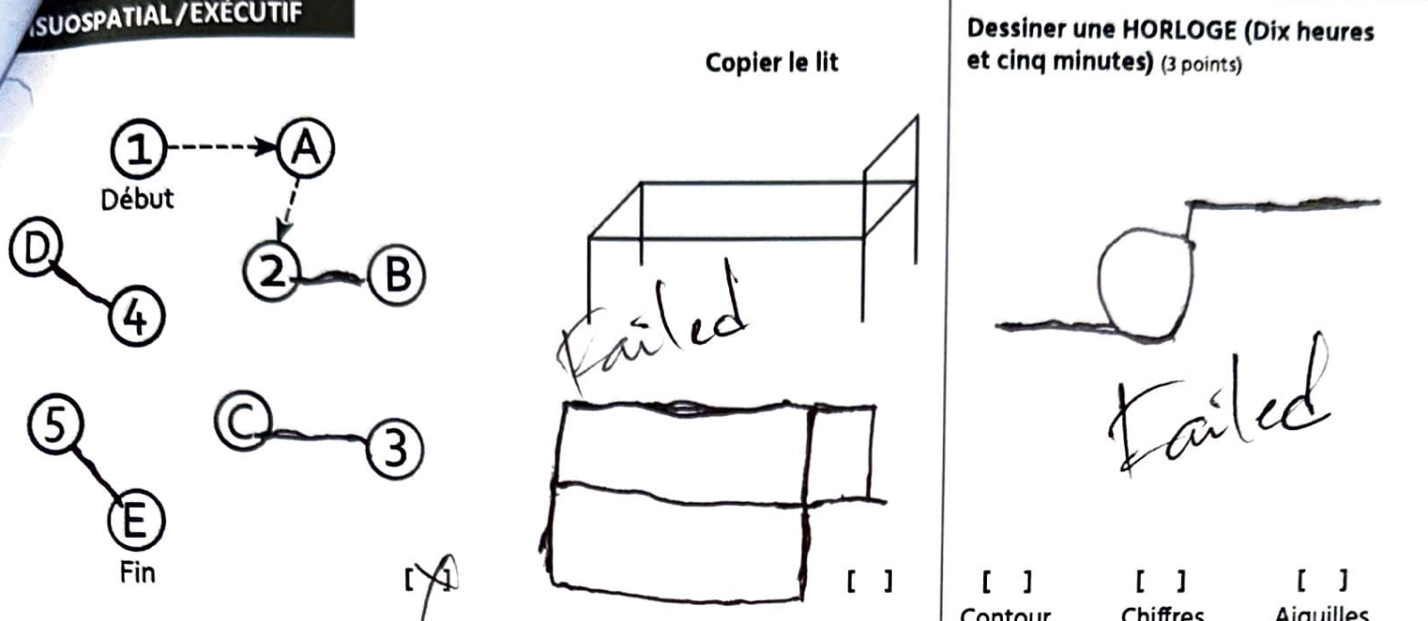


**Appendix III** – Survey Instrument in French

**
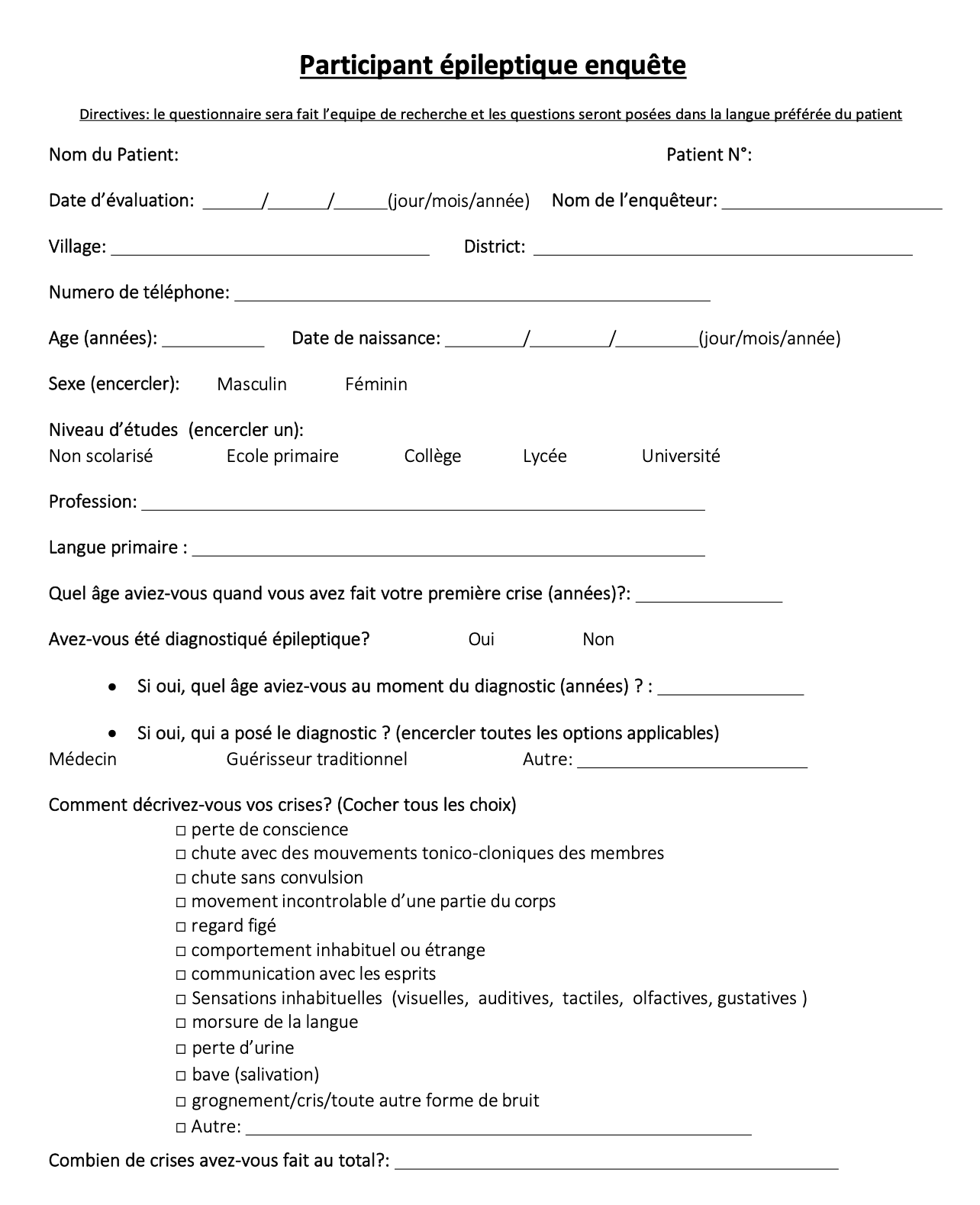
**

**
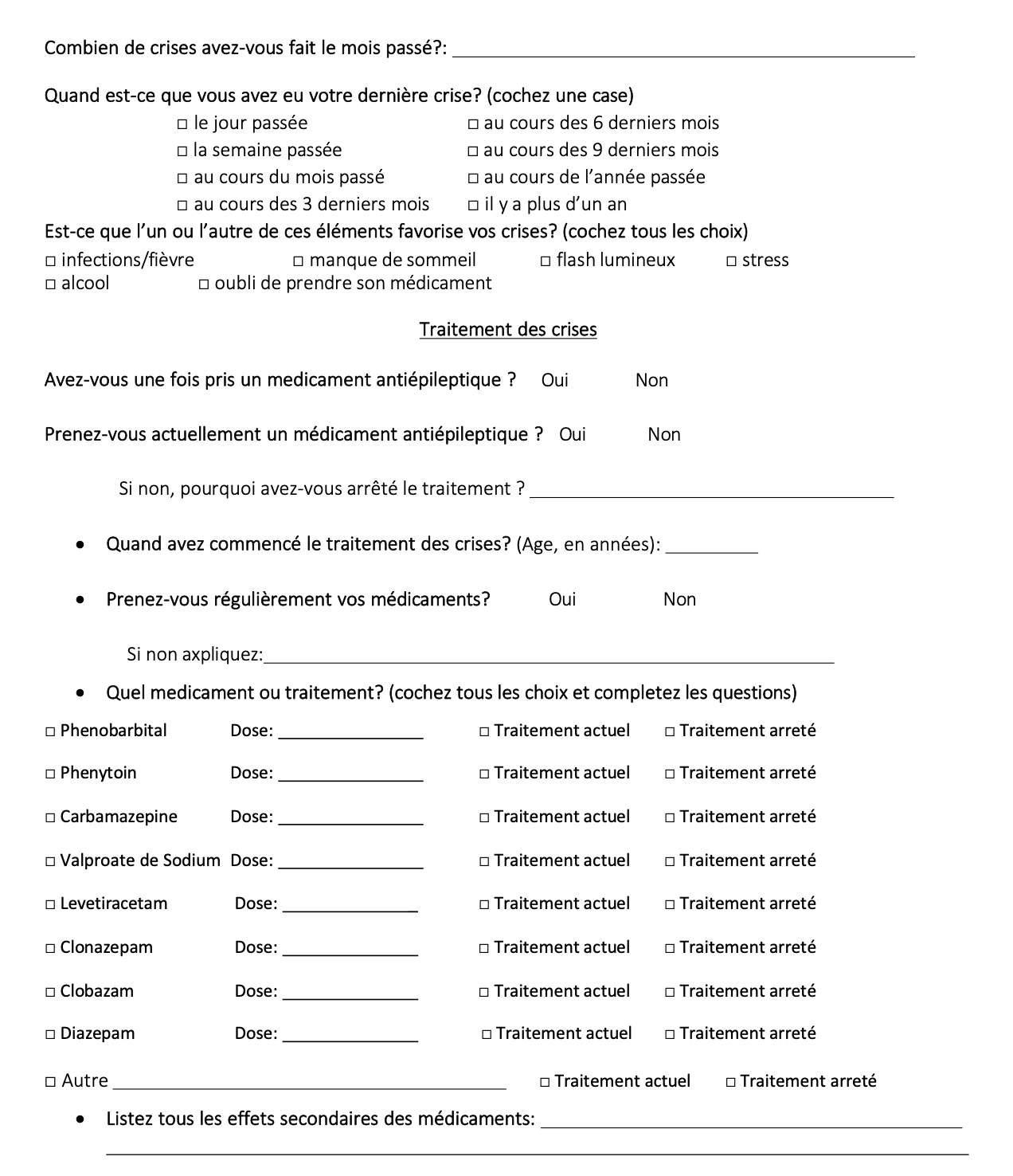

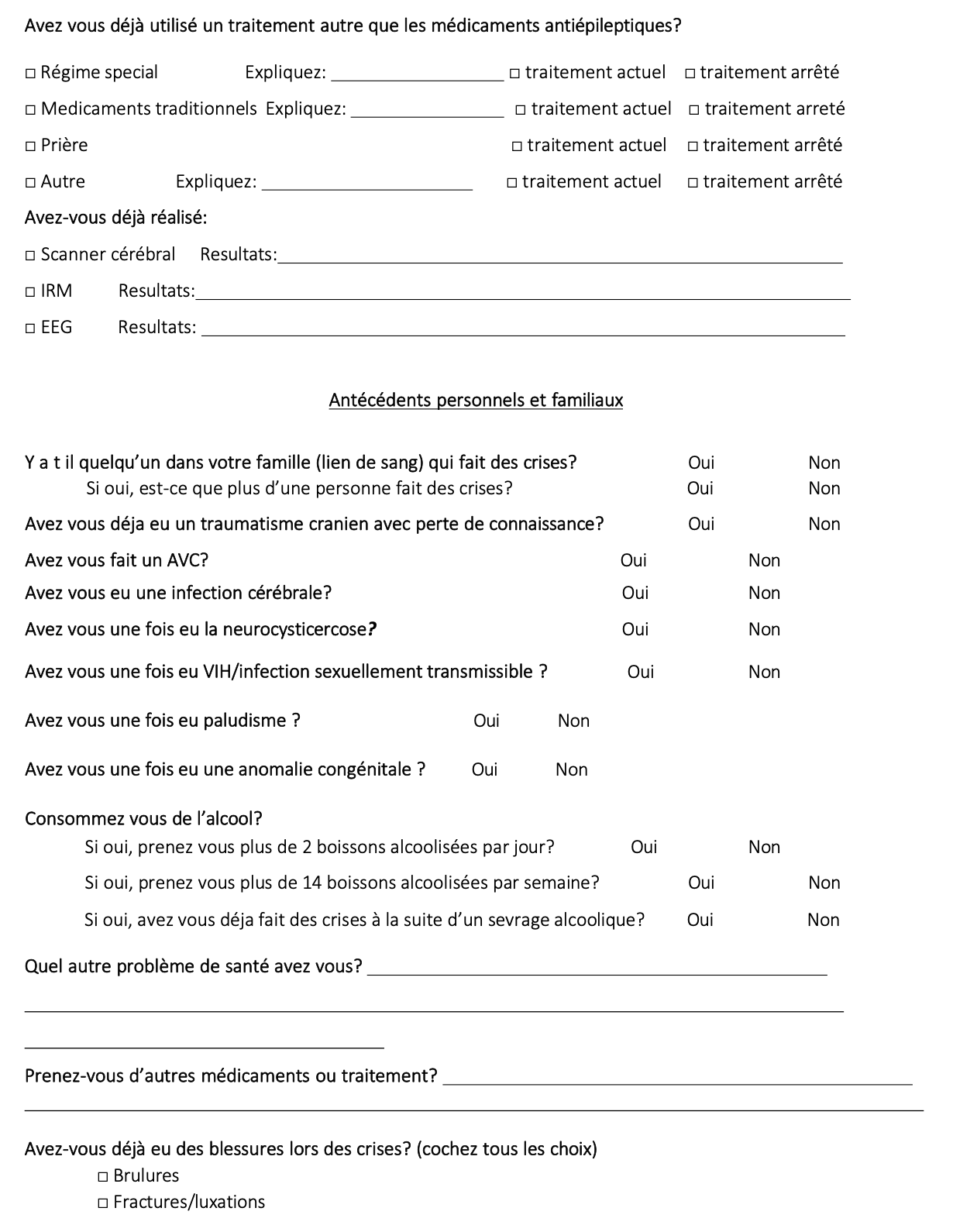

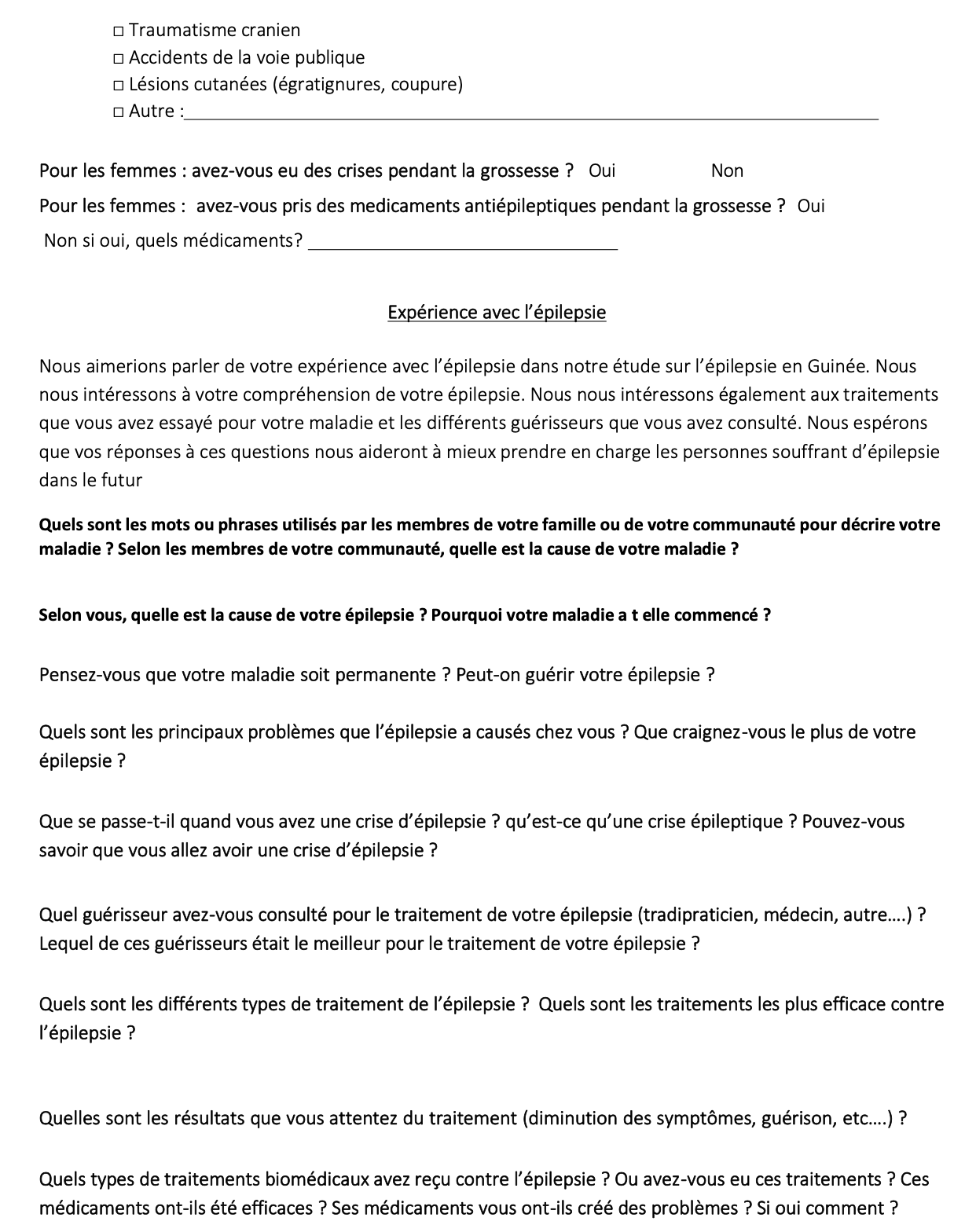
**

**
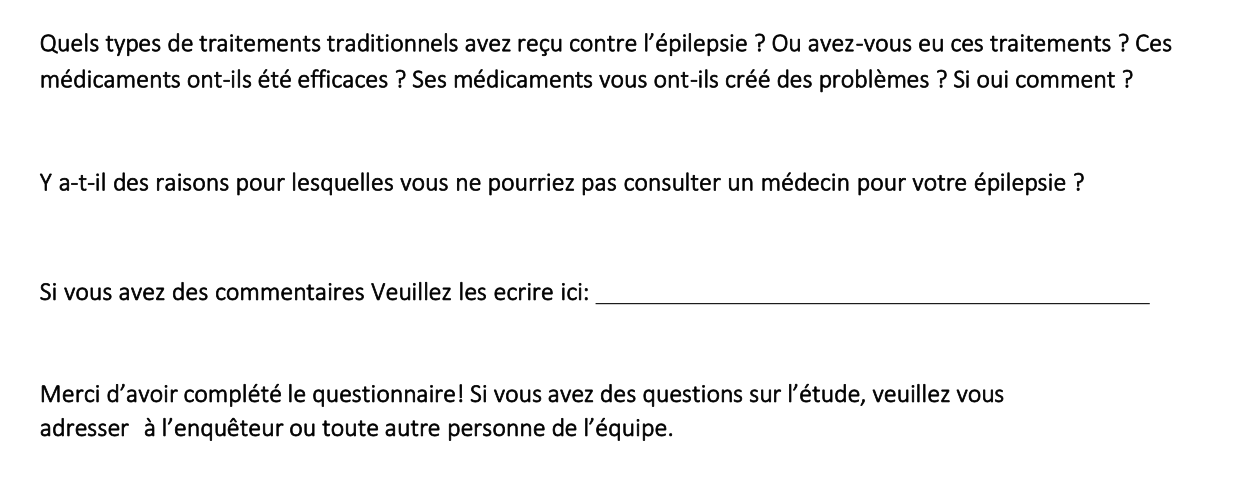
**
